# Effectiveness and safety of dupilumab in the treatment of pediatric atopic dermatitis: a real-world study from China

**DOI:** 10.3389/fimmu.2025.1644875

**Published:** 2025-07-17

**Authors:** Yue Xu, Bowen Li, Wenge Wang

**Affiliations:** ^1^ Graduate School of Hebei North University, Zhangjiakou, Hebei, China; ^2^ Pediatric Department, Air Force Medical Center, PLA, Beijing, China

**Keywords:** dupilumab, pediatric atopic dermatitis, dupilumab-induced paradoxical flare, dupilumab-associated pustular dermatitis, real-world study

## Abstract

**Background:**

Atopic dermatitis (AD), a common chronic inflammatory skin disorder in children, often shows limited response to conventional therapies with potential adverse effects.

**Methods:**

This real-world study evaluated dupilumab—a monoclonal antibody targeting IL-4/IL-13 signaling—in 59 Chinese pediatric patients (aged 6 months–12 years) with moderate-to-severe AD, stratified by body weight. Over a median 33-week follow-up (up to 96 weeks), we dynamically assessed efficacy metrics including Eczema Area and Severity Index (EASI), Peak Pruritus Numerical Rating Scale, and Dermatology Life Quality Index, alongside systematic surveillance of treatment-emergent adverse events (TEAEs).

**Results:**

At Week 16, 68.97% (40/58) achieved EASI-75, accompanied by significant symptom relief (68.17% itch reduction; 77.4% quality-of-life improvement). Efficacy persisted beyond Week 16 (>58.82% sustained EASI-75) without age or sex differences. TEAEs occurred in 25.42% (15/59) of patients, primarily conjunctivitis (10.17%) and paradoxical eczema flares (5.08%). Notably, we report the first pediatric cases of acute rash exacerbation within 72 hours post-initial dose (3 patients, EASI increase: 39.90%–61.13%) and a unique late-onset pustular dermatitis with fever.

**Conclusion:**

These findings confirm dupilumab’s sustained effectiveness and manageable safety in Chinese children with AD while highlighting the need for vigilance against early paradoxical flares and rare inflammatory reactions, providing critical real-world evidence for long-term use in this population.

## 1 Introduction

Atopic dermatitis (AD) represents the most prevalent chronic inflammatory skin disorder in children, with a globally rising incidence. Current epidemiological data indicate that 15–20% of children are affected by AD, and approximately 60% of these patients develop symptoms within the first 12 months of life. This condition imposes a substantial disease burden on the pediatric population (1).

The precise etiology of AD remains incompletely understood. Current management primarily relies on conventional topical therapies, such as corticosteroids and calcineurin inhibitors. However, for children with moderate-to-severe AD, these treatments often yield suboptimal efficacy, frequently necessitating adjunctive systemic interventions (e.g., corticosteroids or immunosuppressants). Nevertheless, long-term use of such systemic agents carries significant safety concerns, particularly in the developing pediatric population. These include potential impacts on growth and development and potential disease recurrence upon discontinuation (2). In contrast, dupilumab offers a promising alternative due to its favorable efficacy and safety profile.

Dupilumab is a monoclonal antibody targeting the interleukin (IL)-4 receptor alpha (IL-4Rα), thereby inhibiting signaling mediated by both IL-4 and IL-13. By blocking IL-4Rα, dupilumab suppresses downstream Th2 inflammatory mediators such as Immunoglobulin E (IgE) and eotaxin-1 (CCL11), modulates immune dysregulation, and interrupts the chronic inflammatory cascade (3). Following its initial approval in China in 2021 for patients aged ≥6 years with moderate-to-severe AD, dupilumab’s indication was extended in 2023 to include children as young as 6 months. It now stands as the only biologic agent approved in China for pediatric moderate-to-severe AD.

Multiple randomized controlled trials (RCTs) have demonstrated dupilumab’s efficacy in significantly improving AD signs, symptoms, and quality of life, alongside a favorable safety profile (4, 5). Nevertheless, real-world evidence regarding its effectiveness and safety in Chinese children remains scarce, with long-term data particularly limited, given its relatively recent introduction to this population.

Therefore, this study aims to explore the actual efficacy and safety profile of dupilumab in Chinese pediatric AD patients through systematic analysis of longitudinal clinical data. We further sought to characterize real-world response profiles and safety patterns, thereby providing region-specific evidence to support its clinical application in pediatric practice.

## 2 Materials and methods

This single-center prospective observational cohort study was conducted at the China Air Force Medical Center from January 2022 to April 2025 to evaluate the real-world effectiveness and safety of dupilumab in pediatric patients with moderate-to-severe AD. The study protocol received approval from the hospital’s Ethics Committee (Approval Number: 2022-28-PJ01). Written informed consent was obtained from all legal guardians, and verbal assent was additionally provided by children aged ≥7 years.

The study protocol planned to include children aged 6 months to 12 years with moderate-to-severe AD, using modified Hanifin & Rajka criteria for children <4 years and Williams criteria for those ≥4 years for diagnosis. All participating physicians completed standardized training in AD diagnosis, encompassing Hanifin & Rajka/Williams criteria and severity grading. Diagnosis and severity classification for every patient underwent independent verification by a second senior physician. One discrepant case was resolved through expert panel arbitration, achieving full consensus.

Disease severity was defined by Eczema Area and Severity Index (EASI) scores: EASI ≥7 to <21 indicated moderate AD, while EASI ≥21 indicated severe AD. Patients with significant systemic comorbidities or active infections were excluded. Dosing regimens were strictly stratified by body weight: children weighing 5 kg to <15 kg received 200 mg subcutaneously every 4 weeks without a loading dose; those weighing ≥15 kg to <30 kg aged under 6 years received 300 mg subcutaneously every 4 weeks without a loading dose. For children aged 6–12 years weighing ≥15 kg to <30 kg, the initial dose was 600 mg (administered as two consecutive 300 mg subcutaneous injections), followed by 300 mg every 4 weeks; those weighing ≥30 kg to <60 kg received an initial 400 mg dose (two consecutive 200 mg injections) followed by 200 mg every 2 weeks; those weighing ≥60 kg received an initial 600 mg dose (two consecutive 300 mg injections) followed by 300 mg every 2 weeks. Efficacy assessments included dynamic monitoring of EASI, itch severity scores, and quality-of-life metrics, with systematic surveillance of treatment-emergent adverse events (TEAEs) throughout the median 33-week follow-up period.

During the baseline period, demographic characteristics, atopic comorbidities, prior treatment history, and disease severity measures were collected. These included the EASI, Investigator’s Global Assessment (IGA), Peak Pruritus Numerical Rating Scale (P-NRS), and quality-of-life scores (≤4 years: Infant’s Dermatitis Quality of Life Index, IDQOL; >4 years: Children’s Dermatology Life Quality Index, CDLQI). Efficacy and safety were evaluated according to a standardized visit schedule at Weeks 2, 4, 8, 12, and 16, followed by assessments every 8–12 weeks thereafter until treatment discontinuation (maximum follow-up: Week 96). At each visit, EASI, IGA, P-NRS, Atopic Dermatitis Control Tool (ADCT) scores, and TEAEs were documented. The primary efficacy endpoint was the percentage change from baseline in EASI score at Week 16. Secondary endpoints included safety metrics (TEAE incidence graded per CTCAE v5.0) and duration of therapy distribution.

By April 30, 2025, 59 pediatric AD patients treated with dupilumab were enrolled. One patient discontinued treatment within 72 hours post-initial dose due to a significant rash exacerbation (EASI increased from baseline 28.9 to 40.2) and was included only in the safety analysis but excluded from efficacy assessments. The remaining 58 patients completed ≥16 weeks of standardized treatment and comprised the final efficacy analysis cohort.

All statistical analyses were performed using SPSS version 27.0 (IBM Corporation, NY, United States) and R version 4.3.3 (R Foundation for Statistical Computing, Vienna, Austria). Quantitative data are expressed as median (interquartile range, IQR), with between-group comparisons analyzed using the Mann-Whitney U test. Categorical data are reported as proportions (%), with intergroup comparisons analyzed using the chi-square test; a P-value < 0.05 was considered statistically significant.

## 3 Result

### 3.1 Clinical demographic characteristics

Patients’ clinical and demographic characteristics are summarized in **Table 1**. This study enrolled 59 pediatric patients aged 2–12 years with moderate-to-severe AD. Baseline assessments revealed severe disease activity (detailed EASI and IGA scores provided in **Table 1**). Baseline serum total IgE levels and eosinophil counts were significantly higher than the upper limit of normal reference values (**Table 1**). Among the 59 patients, 14 (23.72%) failed to respond to previous systemic corticosteroids or immunosuppressants administered before enrollment. Additionally, 40 patients (67.79%) had comorbid atopic conditions including asthma, drug allergy, or food allergy.

**Table 1:** Demographics and clinical characteristics of patients at baseline.

Characteristics	N^a^	Value
Age (years), median (IQR)	59	8.00 (7.00)
Duration of the disease, median (IQR)	59	5.00 (5.00)
IgE, median (IQR)	59	914.49 (1201.20)
Eosinophils, median (IQR)	59	0.57 (0.78)
**Sex**	59	
Female		29 (49.15%)
Male		30 (50.85%)
**Atopic comorbidities**	59	40 (67.79%)
Rhinitis		29 (49.15%)
Asthma		7 (11.86%)
Hives		4 (6.70%)
Food or drug allergy		9 (16.90%)
Contact Dermatitis		1 (1.69%)
**Previous therapy**	59	
Topical corticosteroids		59 (100.00%)
Topical calcineurin inhibitors		28 (47.45%)
Systemic corticosteroids		11 (18.64%)
Methotrexate		3 (5.08%)
JAK kinase inhibitor		2 (3.38%)
Traditional Chinese medicine and Chinese herbal medicine		13 (22.03%)
NB-UVB		6 (10.16%)
**Baseline score**	59	
EASI at baseline, median (IQR)		25.25 (12.85)
IGA at baseline, median (IQR)		4.00 (1.00)
IGA distribution at baseline		
IGA 4		39 (66.10%)
IGA 5		20 (33.90%)
P-NRS at baseline, median (IQR)		8.00 (2.00)
CDLQI at baseline, median (IQR)		15.50 (5.00)
ADCT at baseline, median (IQR)		19.00 (4.00)

Data are n (%) unless otherwise indicated; IgE, immunoglobulin E; NB-UVB, narrowband ultraviolet B; EASI, Eczema Area and Severity Index; IGA, Investigator’s Global Assessment; P-NRS, Pruritus-Numerical Rating Scale; CDLQI, Children Dermatology Life Quality Index; ADCT, Atopic Dermatitis Control Test. ^a^Total number of patients with available data.

### 3.2 Efficacy evaluation

Among the 58 patients included in the efficacy assessment, 28 patients (47.45%) ceased treatment after achieving ≥EASI-75 improvement; three (5.1%) withdrew due to inadequate response; and one (1.7%) discontinued owing to TEAEs. By the final follow-up (median duration: 33 weeks; IQR: 21–53 weeks), 26 patients (44.06%) remained on ongoing therapy.

Efficacy assessments employed a dynamic multi-timepoint monitoring system encompassing baseline evaluations followed by visits at Weeks 2, 4, and 16, with subsequent assessments every 8–12 weeks until treatment cessation, with core metrics including objective signs (EASI, IGA), subjective symptoms (P-NRS), quality-of-life measures (CDLQI for >4 years/IDQOL for ≤4 years), and disease control metrics (ADCT).

**Figure 1:**
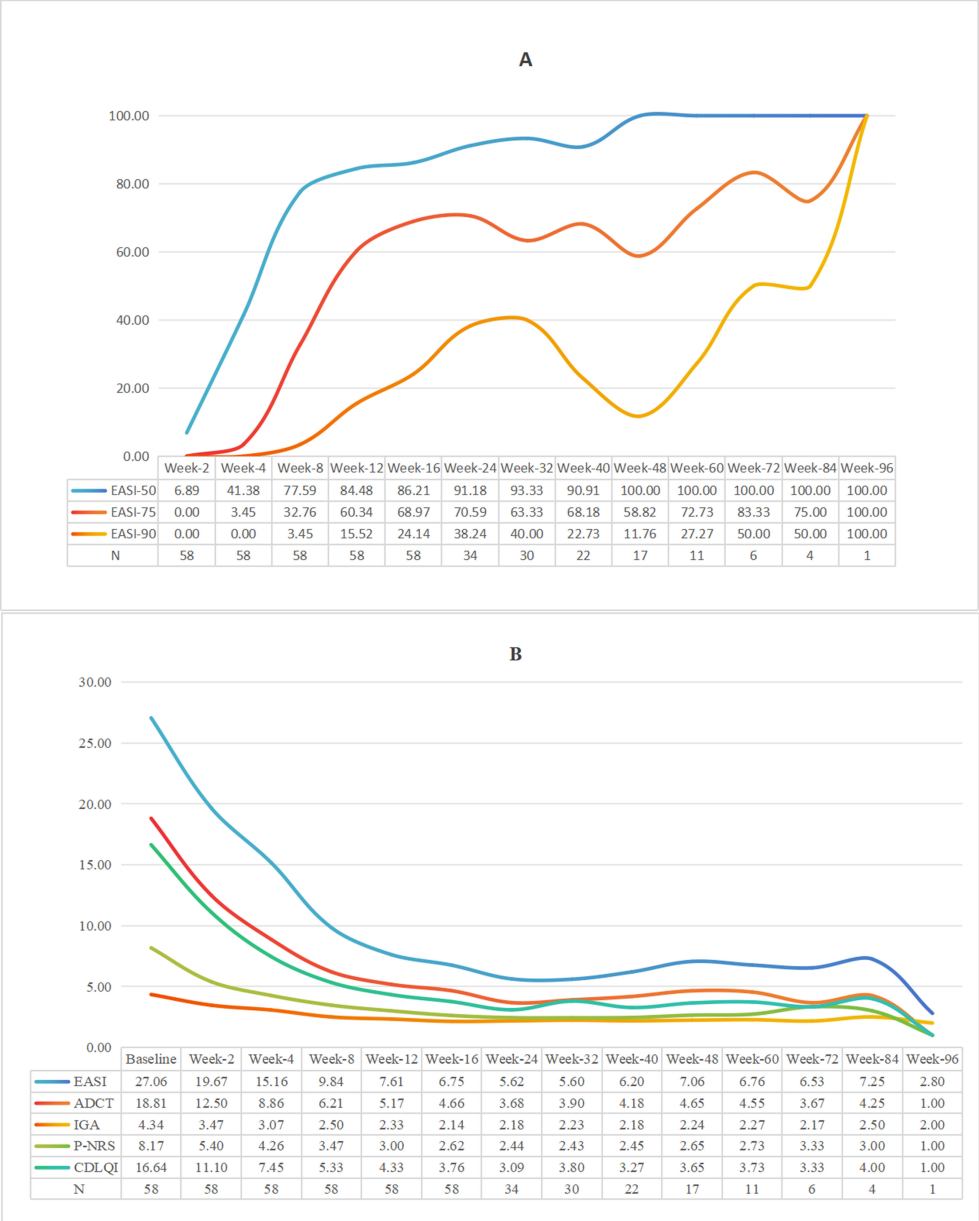
**(A)** The proportion of patients achieving EASl50, 75 and 90 after treatment (%). **(B)** Trend chart of five kinds of score averages over time.

**Figure 2:**
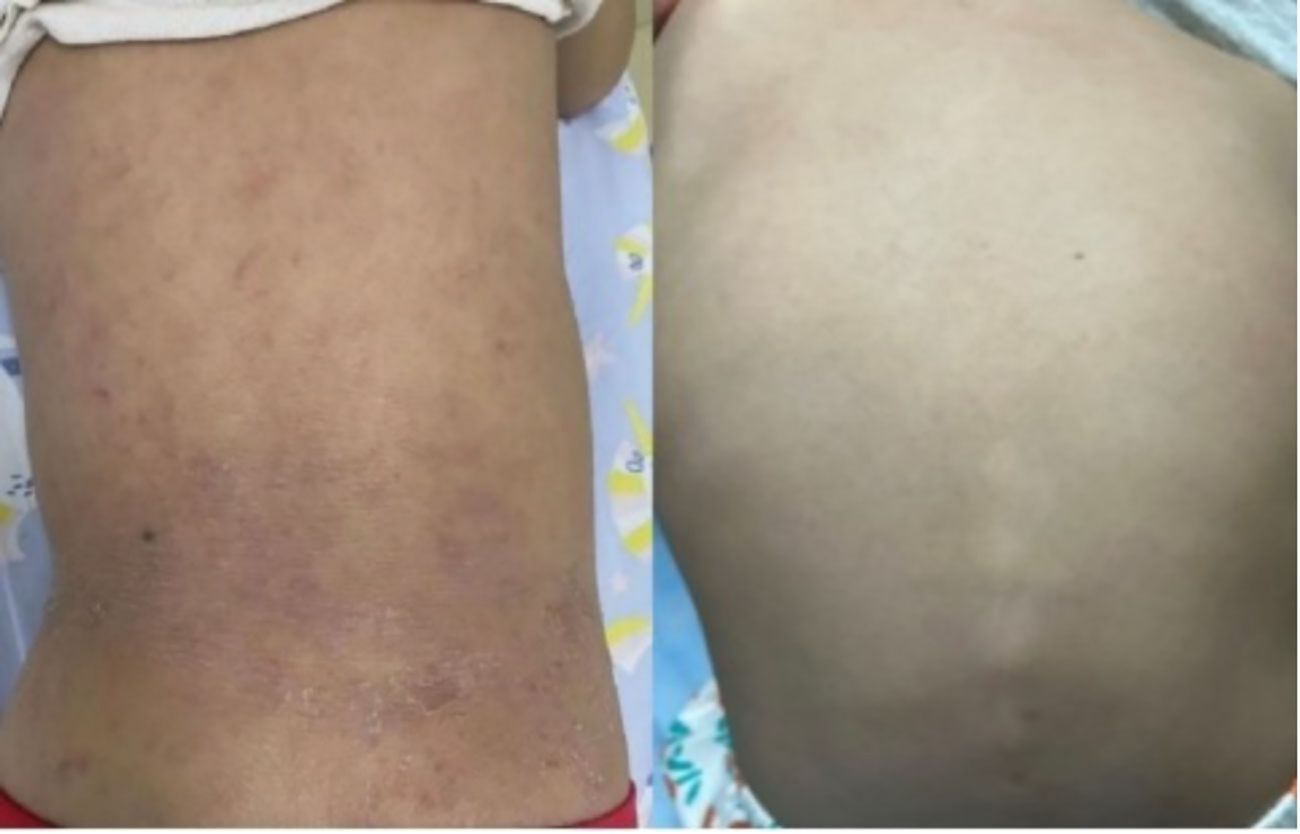
Back skin changes before dupilumab treatment and at 16 weeks of treatment.

Dynamic efficacy assessments among the 58 evaluable patients (one patient excluded from efficacy assessment due to acute rash exacerbation after first dose) showed: at Week 4, EASI-50 response rate was 41.38%; by Week 16, EASI-50, EASI-75 and EASI-90 response rates reached 86.21%, 68.97% and 24.14% respectively (**Figure 1A**); IGA 0/1 achievement rate was 17.24%. Core clinical symptom indicators improved significantly: by Week 16, P-NRS score decreased by 68.17% from baseline, CDLQI/IDLQI score improved by 77.40%, and ADCT score decreased by 75.22% (**Figures 1B**, **2**). During extended treatment (>16 weeks, n=34), EASI-50 response rate consistently exceeded 90%, while EASI-75 response rate ranged between 58.82% and 83.33%.

**Figure 3:**
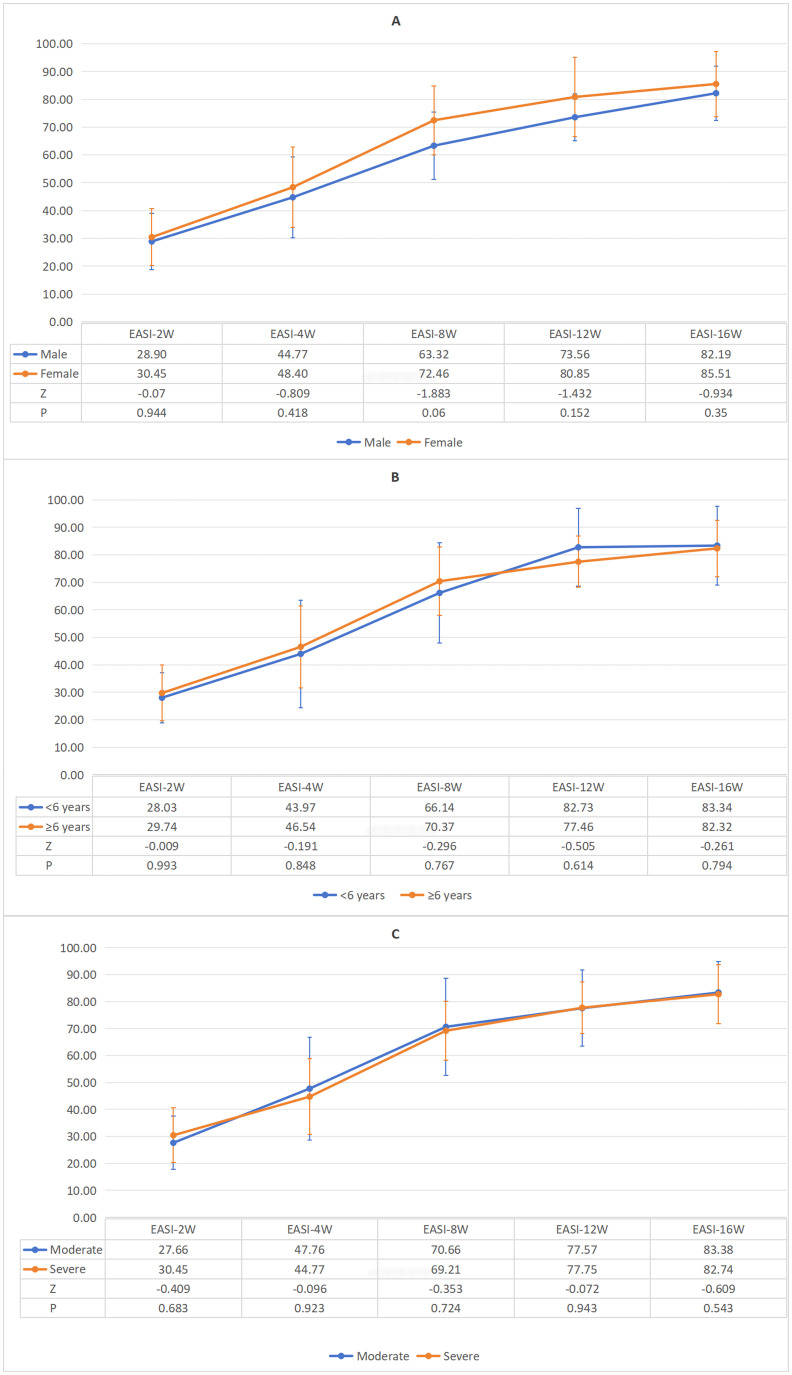
**(A)** Gender-specific variations in mean EASI response rates during standard 16-week therapy. **(B)** Age-specific variations in mean EASI response rates during standard 16-week therapy. **(C)** Severity-specific variations in mean EASI response rates during standard 16-week therapy.

Further subgroup analyses demonstrated that across gender (male and female groups), age (<6 years and ≥6 years subgroups), and disease severity groups—where moderate severity was defined as EASI scores ≥7 to <21 and severe as EASI ≥21—EASI response rates consistently increased over time. No statistically significant differences in EASI response rates were observed between any subgroups at any time points (**Figure 3**). Median EASI score trajectories for all three subgroups are detailed in **Supplementary Figure S1**. These findings suggest that dupilumab’s clinical efficacy is not influenced by gender, age, or baseline disease severity.

**Figure 4:**
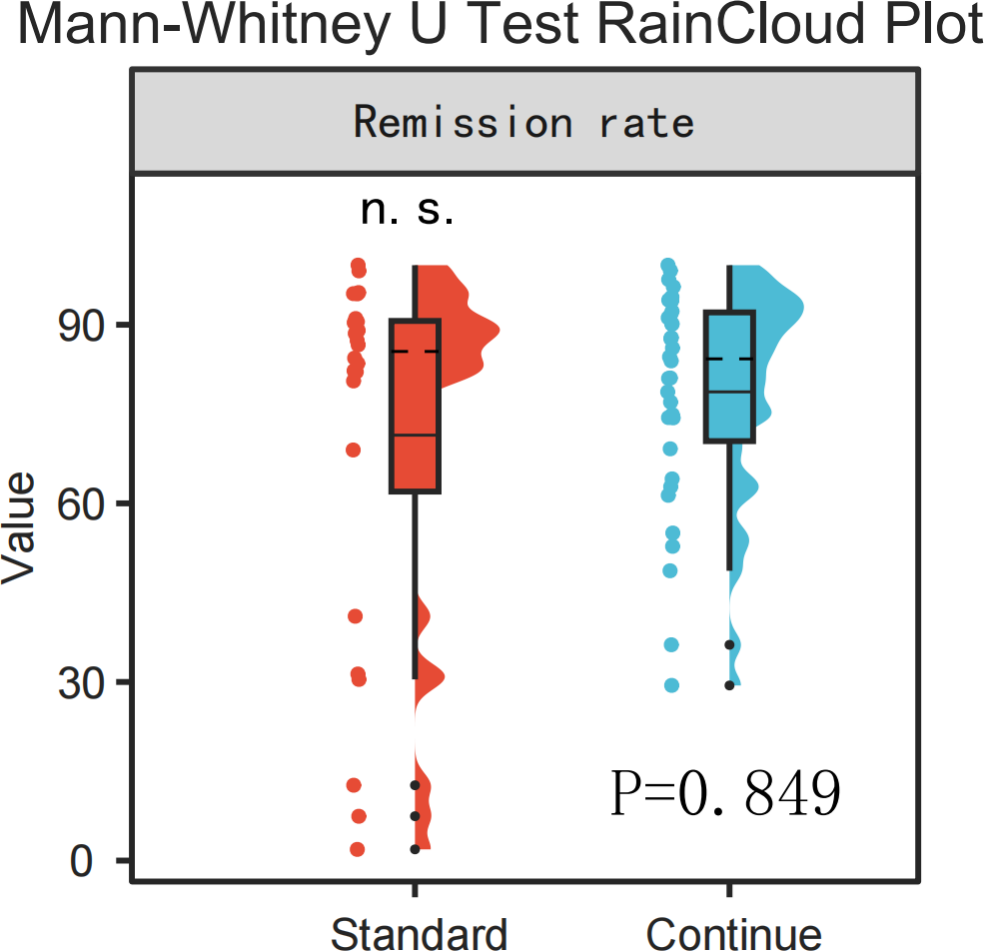
RainCloud plot comparison of remission rates between the standard treatment group and the continued treatment group (Mann-Whitney U Test, p > 0.05).

When comparing response rates at the timepoint endpoint between the standard treatment group (treatment duration =16 weeks, n=24) and the extended treatment group (treatment duration >16 weeks, n=34), no statistically significant intergroup difference was observed (P=0.849), as illustrated in **Figure 4**.

### 3.3 Safety assessment

Systematic safety monitoring was performed for all 59 enrolled patients. A total of 15 children (25.4%) experienced at least one TEAE. The majority of TEAEs were mild to moderate (CTCAE grade 1–2) and exhibited transient characteristics (**Table 2**).

**Table 2:** Adverse events.

	N^a^	Value
Adverse events reported	59	15 (25.42%)
Injection site reactions		2 (3.39%)
Conjunctivitis*		6 (10.16%)
Eye itching		2 (3.39%)
Oral herpes		1 (1.69%)
Joint pain		2 (3.39%)
Vasculitic edema		0 (0%)
Immediate allergic reaction		0 (0%)
Facial rash		0 (0%)
Gastrointestinal reactions		2 (3.39%)
Psoriasis/Psoriasis-like rash		0 (0%)
Increased rash		3 (5.08%)
Papulopustule		1 (1.69%)

Data are n (%), ^a^Total number of patients with available

Data*: Conjunctivitis refers to a group of ocular symptoms, including conjunctivitis, allergic conjunctivitis, keratitis, ulcerative keratitis, blepharitis and dry eye.

Consistent with previous reports, ocular-related adverse events remained the predominant TEAEs, with an incidence of 13.55%. These included 6 cases of conjunctivitis (10.17%) — specifically, 2 cases of allergic conjunctivitis (3.39%) and 4 cases of nonspecific conjunctivitis (6.78%) — as well as 2 cases of ocular pruritus (3.39%). Rash exacerbation constituted the second most common TEAE (incidence: 5.08%), followed by injection-site reactions, gastrointestinal discomfort, and arthralgia (each 3.39%). Oral herpes and pustular rash both exhibited an incidence of 1.69%. Observed gastrointestinal TEAEs primarily manifested as nonspecific symptoms including abdominal pain, nausea, and decreased appetite; no organic lesions were detected on abdominal ultrasound or related examinations.

Additionally, 2 patients (3.39%) developed transient eosinophilia during dupilumab therapy. Absolute eosinophil counts (AECs) increased from 0.27 × 10^9^/L to 0.52 × 10^9^/L at Week 4 (Patient 1) and from 0.50 × 10^9^/L to 1.26 × 10^9^/L at Week 8 (Patient 2). Neither case reached the diagnostic threshold for hypereosinophilia (≥1.5 × 10^9^/L). By Week 16, AECs decreased to 0.32 × 10^9^/L and 0.22 × 10^9^/L respectively, with no associated clinical symptoms.

**Figure 5:**
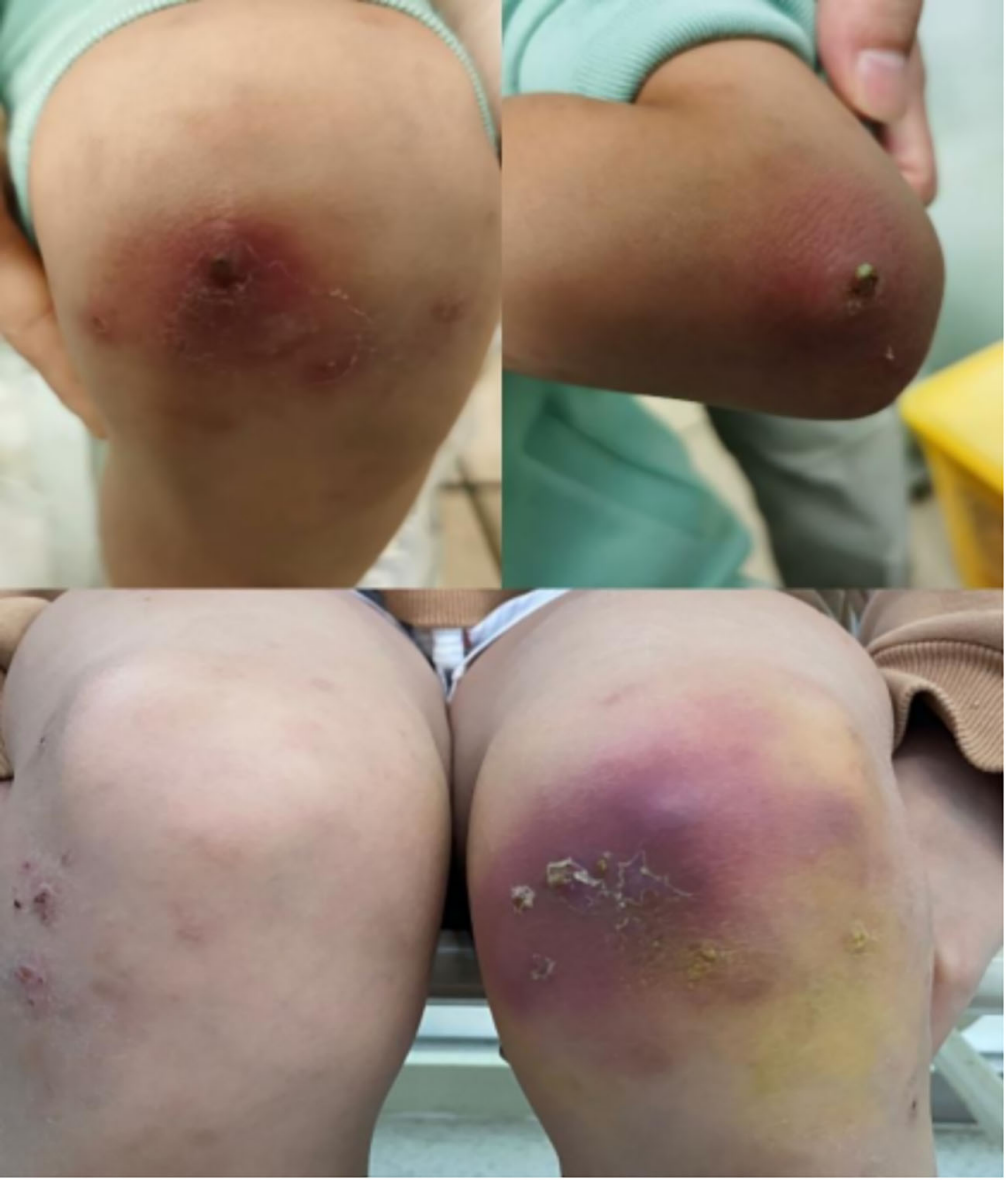
After the 10th treatment with dupilumab, the changes of skin pustular rash in the child: Scattered pustular rash with erythema and scaly crusts could be seen on both elbows. A purple-red patch appears on the extensor side of the right knee joint.

Two treatment-related discontinuations occurred: One child developed acute systemic rash exacerbation within 72 hours post-initial dupilumab administration (EASI increased from 28.9 to 40.2), with symptoms resolving after systemic corticosteroid and topical therapy. The guardians declined further dupilumab treatment. Subsequently, another patient developed pustular dermatitis accompanied by fever and arthralgia following the 10th dose (**Figure 5**). Laboratory findings indicated non infectious inflammation (WBC:7.48×10^9^/L; CRP:0.58mg/L; Procalcitonin: 0.07 ng/mL; ESR: 3 mm/h; Negative HLA-B27 genotyping; Negative antinuclear antibody; Negative pustule fluid culture). AD lesions showed no significant exacerbation. Skin biopsy was declined by guardians, thus the nature of the skin lesion cannot be determined for the time being. Symptoms gradually resolved after dupilumab discontinuation and symptomatic management.

This study documented three cases of dupilumab-associated paradoxical eczema flares. Beyond the previously reported case leading to treatment discontinuation, two additional patients exhibited acute paradoxical flares within 72 hours post-initial dose. These patients demonstrated EASI score increases from 21.8 to 30.5 (Δ=+8.7, +39.91%) and from 24.7 to 39.8 (Δ=+15.1, +61.13%), respectively. After 5–7 days of intervention with high-potency topical corticosteroids combined with emollients, EASI scores decreased to 20.1 and 21.6. Notably, neither patient experienced recurrent flares during subsequent dupilumab therapy, and both maintained ongoing improvement in overall AD manifestations.

Further stratified analyses revealed a significantly higher incidence rate of TEAEs in the severe group compared to the moderate group (35.14% vs. 9.09%, P=0.026). Among gender strata, TEAE incidence rates were 33.33% in males and 17.24% in females (P=0.156). Across age subgroups, rates were 29.41% in the <6-year cohort and 23.81% in the ≥6-year cohort (P=0.906). By treatment duration, incidence was 25.42% in the ≤16-week group and 41.18% in the >16-week group (P=0.114), with no statistically significant differences observed (**Table 3**).

**Table 3:** Comparison of the incidence of TEAEs stratified by gender, age group, treatment time, and disease severity.

	Gender		Age		Treatment Duration		Severity	
	Male	Female	<6 years	≥6 years	Standard	Continue	Moderate	Severe
No (TEAE)	20	24	12	32	44	20	20	24
Yes (TEAE)	10	5	5	10	15	14	2	13
N	30	29	17	42	59	34	22	37
χ^2^	2.014		0.014		2.494		4.936	
P-value	0.156		0.906		0.114		0.026	

Table data are presented as frequencies (n), with row-wise presentation of TEAE occurrence in each subgroup (No = did not occur, Yes = occurred), and column-wise grouping variables (gender, age, treatment duration, Severity). Chi-square test (χ²) was used to assess between-group differences, with P-values derived from two-tailed tests (significance level α = 0.05)

## 4 Discussion

This prospective real-world cohort study evaluated dupilumab in pediatric patients aged 6 months to 12 years with moderate-to-severe AD, with follow-up extending to 96 weeks. As the first biologic agent targeting the IL-4/IL-13 pathway, dupilumab demonstrates substantial efficacy and favorable safety in AD management. In China, where dupilumab has not been widely adopted—particularly for pediatric AD—existing limited domestic studies align with international reports regarding effectiveness. Nevertheless, comprehensive data on long-term treatment remain scarce. Our findings further validate the sustained efficacy of dupilumab in Chinese children with AD (EASI-75 response rate: 68.97% at Week 16; maintained at >58.82% during extended therapy), while also reporting unique safety observations including early-phase paradoxical eczema flares (incidence: 5.08%) and a late-onset pustular dermatitis case. These results augment the real-world safety evidence for dupilumab in clinical practice settings.

Our findings demonstrated superior efficacy outcomes at Week 16 relative to pivotal RCTs, evidenced by an EASI-75 response rate of 68.97% which surpassed rates reported in the *Lancet* Phase 3 trial for children aged ≥6 months (53.0%), a Japanese Phase 3 study (43.3%), and a multinational European/North American clinical trial (46.0%) (4, 6, 7). Cross-comparisons within the real-world evidence landscape revealed: The EASI-75 response rate in our study exceeded that of a small Czech cohort (75.0% at Week 16, n=24) and short-term improvements in a US cohort (mean EASI improvement: 39.6% ± 29.9% at 12–24 weeks, n=89), but fell below a large Spanish study (79.4% at Week 16, n=243). It tended to be comparable to the IGA 0–1 achievement rate in an Israeli cohort at 60 weeks (65.0%, n=230). Within Chinese domestic evidence, our 69.0% response rate was numerically higher than previous Chinese data (63.2%, n=57). Regarding long-term efficacy, the observed >16-week treatment group exhibited an EASI-75 maintenance rate >58%, aligning with sustained improvement patterns in an Italian cohort at 52 weeks (86.8%, n=91) and a Spanish cohort at 52 weeks (85.8%). Notably, subjective symptoms showed remarkable improvement: The reduction in Peak Pruritus Numerical Rating Scale (P-NRS) reached 68.2%, surpassing both the objective sign improvement rate reported in the US cohort (39.6% ± 29.9%) and pruritus relief levels in most European/American RCTs (44.9%–49.4%). This discrepancy may correlate with ethnicity- and geography-dependent itch perception characteristics (8–13). Collectively, these findings demonstrate that dupilumab’s efficacy profile in Chinese pediatric AD patients aligns with the global RCT and real-world evidence framework, while its outstanding subjective symptom control offers a clinically valuable therapeutic option for this population.

At Week 16, most patients achieved significant clinical response (EASI-75). Subgroup analysis revealed no statistically significant difference in response rates between the standard-duration and extended-duration groups (*P*>0.05), suggesting prolonged treatment may not confer additional clinical benefit—a finding contrasting with Paller et al.’s proposition that “extended therapy enhances outcomes” (14). The observed differences in this study may stem from heterogeneity in the enrolled population. Given the relatively small sample size, the statistical power may have been insufficient to detect potential minor clinical benefits. It must be emphasized that “statistical non-significance” does not necessarily equate to “absence of clinical benefit.” Future studies should expand cohorts and incorporate biomarkers to further explore these effects.

During extended follow-up, we observed reduced EASI responses in 3 patients (5.17%) after Week 16 (mean decline: 25.39%). Symptomatic fluctuations during later treatment phases are often attributed to anti-drug antibodies (ADAs); however, existing evidence indicates pediatric ADA incidence is merely 2%–5.3%, typically manifesting as transient low-titer reactions (<1,000), with spontaneous antibody decay occurring during long-term therapy. Consequently, ADA-induced efficacy fluctuations remain rare (15). Notably, reduced frequency of adjunctive topical therapies (particularly emollients) post-symptom improvement was observed in our cohort, potentially contributing to symptom recurrence during dupilumab treatment. Multiple evidence-based studies confirm synergistic effects between dupilumab and topical emollients, aligning with AD’s multifactorial pathogenesis (16). Moreover, American Academy of Dermatology guidelines emphasize “biologics plus topical therapy” as the optimal strategy for sustained disease control (17). Therefore, in clinical practice, symptom fluctuations should not be reflexively ascribed to ADAs, warranting premature discontinuation or dose adjustment. Instead, standardized therapeutic management prioritizing synergistic integration of foundational skincare with biologics is essential to ensure treatment continuity and efficacy.

In this study, treatment discontinuation due to inadequate response occurred in 3 patients (5.08%), lower than the 9.5% non-response rate reported in adult studies (18). This discrepancy may stem from distinct immunophenotypic profiles between pediatric and adult AD. The pathogenesis of AD involves complex immune pathways; certain patients may exhibit dominant non-Th2-mediated inflammation (e.g., Th17 or JAK-STAT activation), potentially limiting dupilumab response (19, 20). Consequently, future research should investigate whether quantitative profiling of pretreatment inflammatory biomarkers (e.g., IL-4, IL-13, IgE) could guide personalized therapeutic strategies, thereby enhancing efficacy while reducing unnecessary drug exposure and associated adverse effects.

Regarding safety, the overall incidence of TEAEs in our study (25.42%) was lower than rates reported in international RCTs (64%–66.7%) (4, 6). Consistent with prior literature, ocular events constituted the primary TEAEs (10.17%), with a moderately higher incidence compared to Western studies—a discrepancy potentially attributable to distinct ocular surface anatomy in East Asian children. We also observed higher AETE risk in children with severe AD, consistent with the studies by Akinlade and Nahum et al.: baseline AD severity is an independent risk factor for dupilumab-associated conjunctivitis (mechanism remains unclear) (21, 22). Although the results are associated with this, it should be noted: smaller sample size may lead to insufficient statistical power (Type II error risk), thus requiring cautious interpretation of this phenomenon.

Additionally, by blocking the IL-4/IL-13 signaling pathway, dupilumab inhibits eosinophil tissue migration, leading to transient retention in peripheral blood. This mechanism may contribute to elevated eosinophil counts and related adverse events (23). However, no hypereosinophilia (≥1.5×10^9^/L) or associated clinical symptoms were observed in our study, consistent with reports by Paller and Wollenberg et al. (incidence: 3.4%, transient nature) (4, 24). Although the vast majority of elevations are benign, asymptomatic events requiring no intervention, long-term therapy warrants vigilance for rare complications (e.g., hypereosinophilic syndrome). Monitoring eosinophil counts during treatment remains essential; such simple laboratory surveillance can effectively prevent severe adverse events and enable proactive risk management.

Notably, this study is the first to systematically document two unique cutaneous reactions associated with dupilumab treatment in pediatric AD: paradoxical eczema flares and incident pustular dermatitis. Three patients (5.08%) exhibited transient paradoxical rash exacerbation during initial treatment, a rarely reported phenomenon in pediatric AD. The mechanism may involve Th1/Th17 immune deviation secondary to Th2 pathway inhibition, whereby IL-4/IL-13 blockade disinhibits the IL-23/IL-17 axis, triggering keratinocyte IL-36β overexpression (25). For the acute pustular rash with fever and arthralgia occurring 12 hours after the 10th dose, the proposed mechanism requires consideration of dupilumab’s microbial modulation effects: The agent achieves rapid *Staphylococcus aureus* clearance (26), but delayed restoration of commensal microbiota equilibrium may promote *Malassezia* colonization dominance (27). Concurrently, IL-13 suppression downregulates antimicrobial peptide expression, compromising cutaneous innate immunity. This dual microbial-immune imbalance state, combined with persistent immune deviation (IL-23/IL-17 pathway activation inducing IL-36γ overexpression), could synergistically trigger acute pustular inflammation. As no skin biopsy was performed, the microbial infiltrate profile and IL-36 pathway activation remain unverified, constituting a significant study limitation.

While mechanistic underpinnings require further investigation, these findings suggest that acute rash exacerbation post-initial dupilumab administration should raise suspicion for transient paradoxical reactions. Furthermore, during extended therapy, vigilant monitoring is warranted for immunomodulation-induced shifts in inflammatory thresholds, which may elevate risk exposure to rare adverse events.

This was a single-center study, which may limit the generalizability of the findings. The limited sample size could also affect the ability to detect rare events. these factors could explain the differences with other studies. Additionally, the lack of detailed comorbidity severity data (e.g., asthma control levels, allergic rhinitis classifications) restricted further understanding of how underlying disease complexity might influence dupilumab treatment outcomes. Future prospective studies should comprehensively evaluate dupilumab application in children by incorporating objective comorbidity severity measures and expanding sample sizes, with a focus on addressing unresolved challenges in real-world pediatric AD management, including: tracking the long-term impacts of dupilumab beyond 96 weeks of therapy on child growth, development, and other organ systems via multicenter large-scale cohorts; elucidating the mechanisms by which ADA seropositivity contributes to reduced efficacy through ADA testing and trough concentration analysis; developing predictive models that integrate inflammatory cytokine profiles (e.g., IL-4, IL-13, IL-22) and microbiome signatures (e.g., *Malassezia* colonization), with prospective validation of their utility in predicting treatment response and specific adverse events; ultimately enabling safer and more effective personalized treatment strategies for children with AD.

## 5 Conclusion

Dupilumab demonstrated significant efficacy and favorable safety in Chinese pediatric patients (aged 6 months to 12 years) with moderate-to-severe AD. At Week 16, the EASI-75 response rate reached 68.97%, with a long-term maintenance rate exceeding 58.82%. Unlike previous reports, this study showed no additional improvement in response rates for children treated >16 weeks compared to those receiving 16 weeks of standard therapy; larger sample sizes are needed to confirm this observation. Furthermore, this study is the first to systematically document early-treatment paradoxical rash exacerbation (incidence: 5.08%) and rare *de novo* pustular rash (1.69%), highlighting the clinical relevance of these special events. Ocular adverse events constituted the most common mild-to-moderate side effects (13.56%). These findings provide crucial locally generated evidence for dupilumab’s clinical application in Chinese children with AD and supplement surveillance data on unique adverse drug reactions in this pediatric population.​

## Data Availability

The raw data supporting the conclusions of this article will be made available by the authors, without undue reservation.
