# Supplementary material for: Effectiveness and safety of dupilumab in the treatment of pediatric atopic dermatitis: a real-world study from China
Source: Front Immunol. 2025 Jul 17;16:1644875. doi: 10.3389/fimmu.2025.1644875 (PMC12310642; doi:10.3389/fimmu.2025.1644875)
Supplement: Supplementary file 1 [file DataSheet1.docx]

**Supplementary Figure S1**

**
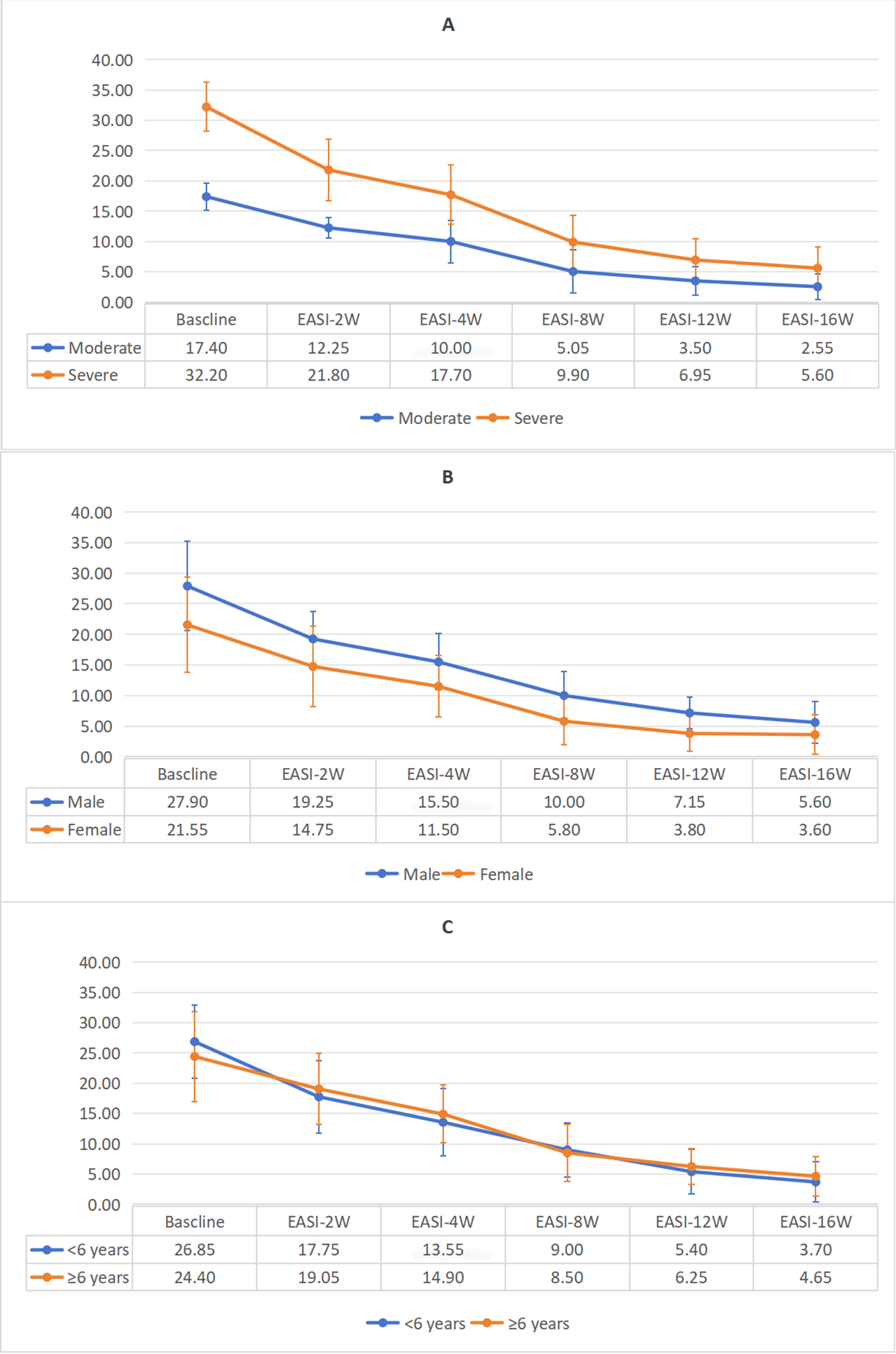
**

Supplementary Figure S1：Longitudinal Changes in Median EASI Scores (Interquartile Ranges) Stratified by Subgroups.
